# Research on the Prediction of Driver Fatigue Degree Based on EEG Signals

**DOI:** 10.3390/s25237316

**Published:** 2025-12-01

**Authors:** Zhanyang Wang, Xin Du, Chengbin Jiang, Junyang Sun

**Affiliations:** School of Electrical Engineering, Beijing Jiaotong University, Beijing 100044, China; 23126367@bjtu.edu.cn (Z.W.); 23126290@bjtu.edu.cn (C.J.); 23126348@bjtu.edu.cn (J.S.)

**Keywords:** electroencephalogram, CTL-ResFNet, driver fatigue prediction, deep learning, PERCLOS

## Abstract

Objective: Predicting driver fatigue degree is crucial for traffic safety. This study proposes a deep learning model utilizing electroencephalography (EEG) signals and multi-step temporal data to predict the next time-step fatigue degree indicator percentage of eyelid closure (PERCLOS) while exploring the impact of different EEG features on prediction performance. Approach: A CTL-ResFNet model integrating CNN, Transformer Encoder, LSTM, and residual connections is proposed. Its effectiveness is validated through two experimental paradigms, Leave-One-Out Cross-Validation (LOOCV) and pretraining–finetuning, with comparisons against baseline models. Additionally, the performance of four EEG features—differential entropy, α/β band power ratio, wavelet entropy, and Hurst exponent—is evaluated, using RMSE and MAE as metrics. Main Results: The combined input of EEG and PERCLOS significantly outperforms using PERCLOS alone validated by LSTM, and CTL-ResFNet surpasses baseline models under both experimental paradigms. In LOOCV experiments, the α/β band power ratio performs best, whereas differential entropy excels in pretraining–finetuning. Significance: This study presents a high-performance hybrid deep learning framework for predicting driver fatigue degree and reveals the applicability differences in EEG features across experimental paradigms, offering guidance for feature selection and model deployment in practical applications.

## 1. Introduction

Fatigued driving is a condition in which a driver’s driving skills deteriorate after driving a vehicle for an extended period of time [1]. Driver fatigue can be caused by poor sleep quality, prolonged driving of vehicles, single road conditions, and taking medications prohibited for driving vehicles. Continuing to drive while fatigued is highly dangerous and may lead to traffic accidents. Timely detection of the driver’s drowsiness can help prevent accidents caused by fatigue. Fatigue-related traffic accidents often result in catastrophic outcomes, including multiple fatalities and injuries [2]. An analysis of rail accidents in the UK shows that about 21% of high-risk rail accidents are caused by driver fatigue [3]. The above figures show that fatigue driving is not only common but also has serious consequences, thus demonstrating the importance of preventing fatigue driving.

Currently, research on fatigue driving detection primarily focuses on binary classification (fatigued or alert) or ternary classification (alert, fatigued, and drowsy) of the driver’s fatigue state [4,5,6,7,8,9]. However, these approaches have notable limitations. First, classification models can only passively identify fatigue states that have already occurred, lacking the capability to dynamically predict the evolution of fatigue. Second, traditional methods often rely on single-modal signals, such as eye movements or facial features, making them inadequate to address individual differences and environmental interference in complex driving scenarios. To address these issues, this paper proposes a multimodal temporal prediction framework, CTL-ResFNet, which integrates multidimensional dynamic features such as eye state and EEG signals. This framework achieves a paradigm shift from state recognition to fatigue trend prediction, providing a novel technical approach for proactive fatigue intervention.

The main contributions of this paper include:

(1) The CTL-ResFNet hybrid neural network is proposed, which for the first time organically integrates the local feature extraction capability of CNN, the global dependency modeling of Transformer, and the temporal dynamic capturing of LSTM through residual connections, addressing the representational limitations of traditional single models in cross-modal EEG-fatigue temporal prediction. Experiments demonstrate that this architecture significantly outperforms baseline models in both leave-one-out cross-validation (LOOCV) and transfer learning scenarios, providing a new paradigm for physiological signal temporal prediction.
(2) Through LOOCV, it was discovered that the spatiotemporal coupling of EEG differential entropy features and PERCLOS can improve prediction accuracy, revealing the complementary enhancement effect of physiological signals on subjective fatigue labels. Further research revealed that in LOOCV, the predictive performance of the α/β band energy ratio significantly outperforms comparative features such as differential entropy and wavelet entropy, demonstrating its superior zero-shot cross-subject generalization ability and stronger robustness to individual differences.
(3) To validate the small-sample individual adaptation capability of CTL-ResFNet, this study established a pretraining–finetuning experimental framework. The results demonstrate that differential entropy features exhibit optimal performance in transfer learning scenarios. This finding provides methodological guidance for optimizing feature selection in practical fatigue monitoring system applications.

This study focuses on the prediction of driver fatigue degree based on EEG signals. The paper is divided into five sections: Section 1 elaborates on the hazards of fatigued driving and the significance of the research, presenting the research questions. Section 2 systematically reviews existing fatigue detection methods and summarizes related work. Section 3 introduces the proposed CTL-ResFNet hybrid neural network architecture, which integrates the advantages of CNN, Transformer, and LSTM while incorporating residual connections. Section 4 presents the experimental validation, including a detailed description of the SEED-VIG dataset, experimental setup, evaluation metrics, and comparative experiments. Section 5 analyzes the results and discusses the effectiveness of different EEG features in predicting fatigue degree.

## 2. Related Work

In the field of driver fatigue detection, numerous scholars have conducted extensive research based on EEG signals. Early studies primarily focused on the correlation between EEG rhythmic waves and fatigue states. For instance, researchers observed that as fatigue levels in-creased, the power of α and θ waves significantly rose [10,11], while the power of β waves exhibited a declining trend [12]. Subsequently, Jap et al. reported that the (θ+α)/β ratio showed the most pronounced increase at the end of driving tasks [13], which aligns with the conclusions of earlier studies. These findings laid a crucial foundation for subsequent research, prompting scholars to systematically explore the quantitative relationship between EEG activities in different frequency bands and fatigue levels.

With the advancement of machine learning techniques, researchers gradually shifted from traditional spectral analysis to more sophisticated feature extraction and pattern recognition methods. For example, Khushaba et al. employed wavelet packet transform based on fuzzy mutual information for feature extraction, achieving an average classification accuracy of 95–97% for driver fatigue states [14]. Zhao et al. utilized a multivariate autoregressive model to extract features from multi-channel EEG signals, combined with kernel principal component analysis and support vector machines, attaining an identification accuracy of 81.64% for three driving-related mental fatigue states [15]. In addition, some scholars have explored fatigue detection by integrating multimodal physiological signals. For instance, Huo et al. fused EEG and frontal electrooculogram (EOG) signals and applied a discriminant graph regularized extreme learning machine to detect driver fatigue levels. Their results demonstrated that the fused modality outperformed single-modality signals in fatigue detection performance [16].

In recent years, the rise of deep learning methods has brought new breakthroughs in fatigue detection. For example, Chaabene et al. achieved a high accuracy of 90.42% in fatigue state classification based on EEG signals and a convolutional neural network architecture [17]. Cui et al. utilized separable convolutions to process spatiotemporal sequences of EEG signals, reporting an average accuracy of 78.35% in LOOCV across 11 participants [18]. In terms of feature engineering, some researchers have begun exploring the application of nonlinear dynamic features. Shi et al. proposed a novel EEG feature called differential entropy in their study [19], with experimental results demonstrating that differential entropy (DE) is the most accurate and stable EEG feature for reflecting vigilance changes. Notably, most existing fatigue detection methods focus on identifying fatigue after it has already occurred, lacking the capability for dynamic prediction of fatigue degree, which provides a critical research opportunity for this study.

## 3. Methods

### 3.1. Overall Architecture

**Figure 1:**
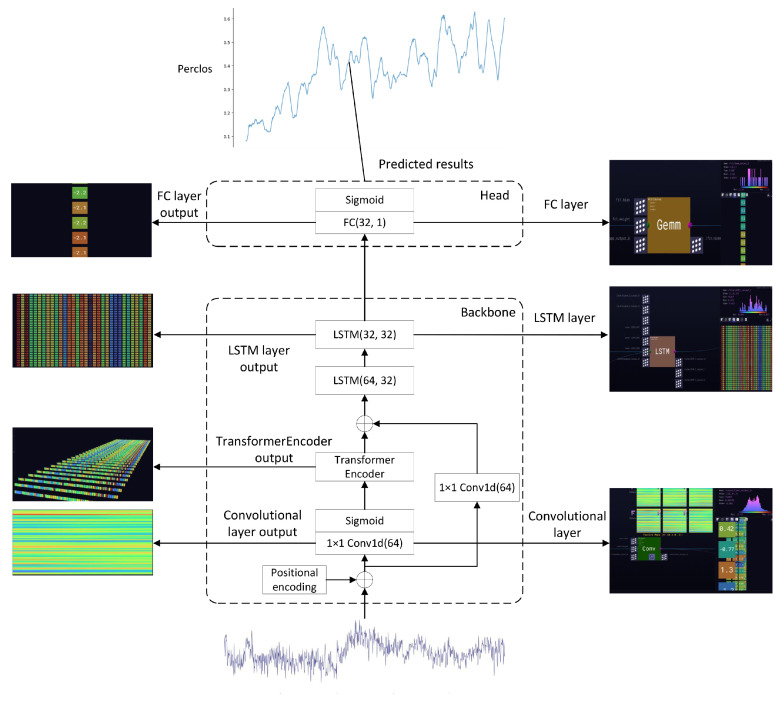
CTL-ResFNet Model overall structure.

The proposed EEG-based fatigue degree prediction model consists of two core components: a feature extraction backbone network and a prediction regression head. The backbone network integrates CNN, Transformer encoder, LSTM, and residual networks, while the regression head comprises a fully connected layer with an output dimension of 1. The overall architecture is illustrated in Figure 1.

The model employs a CNN for dimensionality reduction in input data, utilizes the Transformer encoder architecture to capture diverse features and relationships within sequences, and subsequently feeds the extracted feature sequences into an LSTM network to extract temporal dependencies. Furthermore, residual connections are incorporated to enhance information flow and mitigate gradient vanishing issues. Finally, a fully connected layer performs regression prediction of fatigue degree. The model parameters are shown in the following table (Table 1).

To provide a theoretical rationale, the CTL-ResFNet is designed according to a “Spatial-Global-Temporal” processing hierarchy tailored to EEG characteristics. First, the CNN employs 1×1 convolutions as learnable spatial filters. Unlike temporal convolutions, this design deliberately fuses cross-channel information to simulate neural integration across cortical regions while strictly preserving the temporal resolution of the sequence. Second, the Transformer Encoder models global functional connectivity, capturing long-range dependencies between distant electrodes that local operations miss. A single-layer configuration was adopted to prevent overfitting, balancing model capacity with the limited sample size of the EEG dataset. Third, the LSTM layer captures the cumulative temporal evolution of fatigue, treating drowsiness as a dynamic process accumulated over time. Finally, residual connections ensure training stability against the high variability inherent in physiological signals.

### 3.2. Position Encoding and Activation Function

Since the Transformer encoder does not inherently capture positional information, sinusoidal positional encoding is employed:

(1) $$PE_{\left(pos,2i\right)}=\sin\left(\frac{pos}{{10,000}^{2i/d_{model}}}\right)$$

(2) $$PE_{\left(pos,2i+1\right)}=\cos\left(\frac{pos}{{10,000}^{2i/d_{model}}}\right)$$

where pos denotes the input position, $d_{model}$ is the feature dimension, and *i* the feature index.

The model uses two nonlinear activation functions: ReLU and Sigmoid. ReLU is defined as

(3) f(x)=max(0,x)

which maintains a gradient of 1 for positive inputs, mitigating gradient vanishing in deep networks.

The Sigmoid function,

(4) $$f\left(x\right)=\frac{1}{1+e^{-x}}$$

outputs values in (0,1), making it suitable for probability prediction.

### 3.3. CNN Module

CNN is adopted due to its strong capability for extracting local spatial correlations in multichannel EEG data, which helps to capture frequency-specific spatial patterns related to fatigue. Compared with traditional feature engineering or simple MLP, CNN can automatically learn discriminative spatial filters from EEG inputs [20]. Convolutional Neural Networks were first proposed by Yann LeCun et al. [21] in 1998, primarily for image processing. CNNs have since been extended to handle one-dimensional data, such as time series. In this study, we employ a one-dimensional CNN (1D CNN) architecture, where convolution kernels slide along the input sequence to compute dot products of local regions.

**Figure 2:**
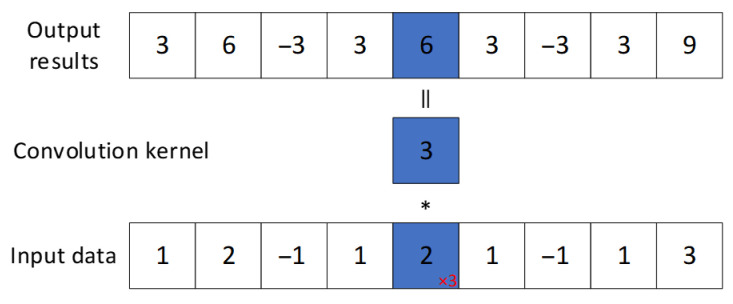
1D CNN (kernel_size = 1).

**Figure 3:**
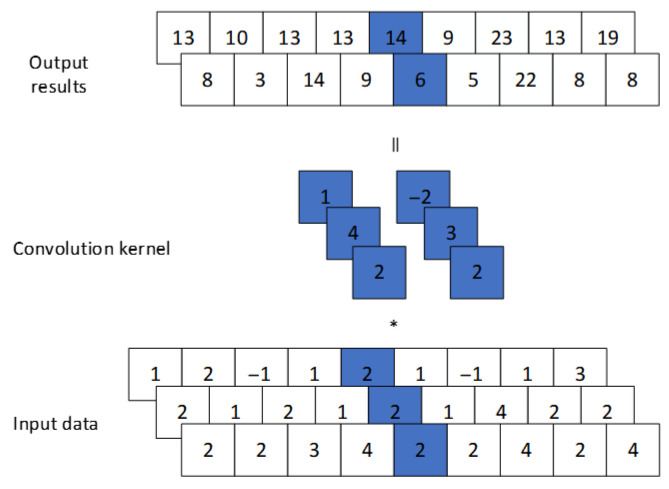
1D CNN (kernel_size = 1, in_channels = 3, out_channels = 2).

Figure 2 illustrates the 1×1 convolutional kernel used in this study. It operates on individual elements of the input data, producing output with the same spatial dimensions but cannot capture relationships between adjacent elements. This operation is applied to multi-channel input for dimensionality reduction: as shown in Figure 3, a 1D convolution with size 1 transforms a 3-channel input into a 2-channel output without altering sequence length.

### 3.4. Transformer Encoder Module

**Figure 4:**
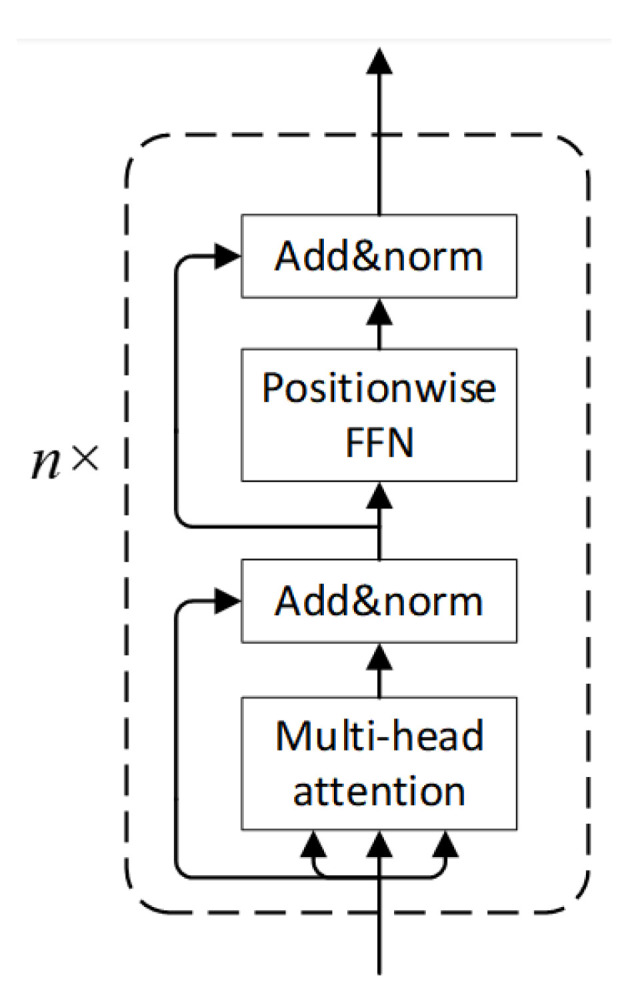
Transformer Encoder.

In 2017, Vaswani et al. [22] introduced the Transformer architecture, consisting of an encoder and a decoder. In this study, only the Transformer encoder is employed, as shown in Figure 4.

The Transformer encoder is composed of *n* stacked layers (n=1 in this study), each containing two sub-layers: a multi-head self-attention mechanism followed by layer normalization and residual connection, and a position-wise feed-forward network with similar normalization and residual structure.

The scaled dot-product attention is defined as:

(5) $$Attention\left(Q,K,V\right)=softmax\left(\frac{QK^{T}}{\sqrt{d_{k}}}\right)V$$

where *Q*, *K*, and *V* are query, key, and value vectors, and $d_{k}$ is the key dimension.

Multi-head attention enables capturing diverse feature representations:

(6) $$\begin{array}{c} Q_{i}=XW_{i}^{Q},\;K_{i}=XW_{i}^{K},\;V_{i}=XW_{i}^{V} \end{array}$$

(7) $$\begin{array}{c} head_{i}=\mathrm{Attention}\left(Q_{i},K_{i},V_{i}\right) \end{array}$$

(8) $$\begin{array}{c} \mathrm{MultiHead}\left(Q,K,V\right)=\mathrm{Concat}\left(head_{1},\ldots ,head_{h}\right)W^{O} \end{array}$$

where $W_{i}^{Q},W_{i}^{K},W_{i}^{V},W^{O}$ are learnable weights.

The feed-forward network enhances model capacity:

(9) $$f\left(x\right)=\max\left(0,xW_{1}+b_{1}\right)W_{2}+b_{2}$$

Residual connections [23] mitigate vanishing/exploding gradients:

(10) H(x)=F(x)+x

Layer normalization stabilizes training by reducing internal covariate shift, normalizing inputs across channels.

### 3.5. LSTM Module

Long Short-Term Memory (LSTM), proposed by Hochreiter and Schmidhuber in 1997 [24], is a variant of traditional RNNs with superior ability to capture long-range dependencies in sequential data. LSTM architectures have been successfully applied to fatigue detection tasks [25].

**Figure 5:**
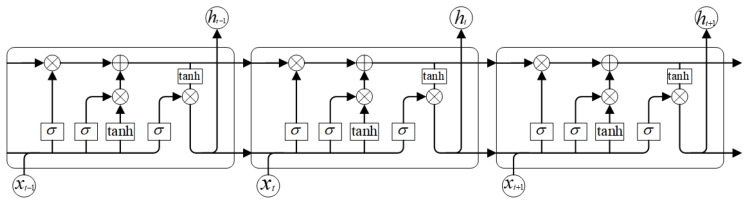
LSTM structure.

An LSTM unit relies on four key components: the forget gate, input gate, cell state, and output gate. Figure 5 illustrates the internal structure of a single LSTM unit. At timestep *t*, the unit receives the input $x_{t}$ and the previous hidden state $h_{t-1}$, producing an updated cell state $C_{t}$ and hidden state $h_{t}$, which are then propagated to the next timestep.

The forget gate decides which historical information to discard:

(11) $$f_{t}=\sigma \left(W_{f}\cdot \left[h_{t-1},x_{t}\right]+b_{f}\right)$$

The input gate determines how much new information to store:

(12) $$i_{t}=\sigma \left(W_{i}\cdot \left[h_{t-1},x_{t}\right]+b_{i}\right)$$

(13) $${\tilde{C}}_{t}=\tanh\left(W_{C}\cdot \left[h_{t-1},x_{t}\right]+b_{C}\right)$$

The cell state is updated as:

(14) $$C_{t}=f_{t}*C_{t-1}+i_{t}*{\tilde{C}}_{t}$$

The output gate controls the hidden state output:

(15) $$o_{t}=\sigma \left(W_{o}\cdot \left[h_{t-1},x_{t}\right]+b_{o}\right)$$

(16) $$h_{t}=o_{t}*\tanh\left(C_{t}\right)$$

This simplified structure illustrates how LSTM selectively retains or discards information, enabling effective modeling of long-range dependencies in sequential data.

### 3.6. Regression Prediction Head

The hidden state output from the final timestep of the LSTM layer in the feature extraction backbone network is connected to the regression prediction head. The regression head consists of a fully connected (FC) layer with an input dimension of 32 and an output dimension of 1. Since the output values are constrained within the range (0,1), a sigmoid activation function is employed.

## 4. Experiments

### 4.1. SEED-VIG Dataset

EEG signals represent the scalp-recorded electrical potentials generated by synchronous discharges of neuronal populations, primarily originating from the synchronized synaptic activities of pyramidal cells in the cerebral cortex [26]. Specifically, the human brain contains tens of thousands of interconnected neurons. These neurons receive signals from other neurons and generate EEG signals when the received signals exceed a certain threshold. Essentially, brain waves represent the electrical signals produced by the collective activity of these neurons. Single-channel EEG signals provide limited information with poor determinacy, leading to random research outcomes, whereas multi-channel EEG signals can capture more comprehensive information and better reflect global brain activities. EEG signals possess inherent non-replicability as physiological characteristics. Relevant studies have demonstrated significant differences in EEG patterns between fatigue and non-fatigue states [27]. Among various physiological indicators for fatigue assessment, EEG signals are recognized as one of the most reliable biomarkers [28]. Researchers have made notable breakthroughs in monitoring fatigue levels by leveraging EEG signal analysis [29,30,31].

**Figure 6:**
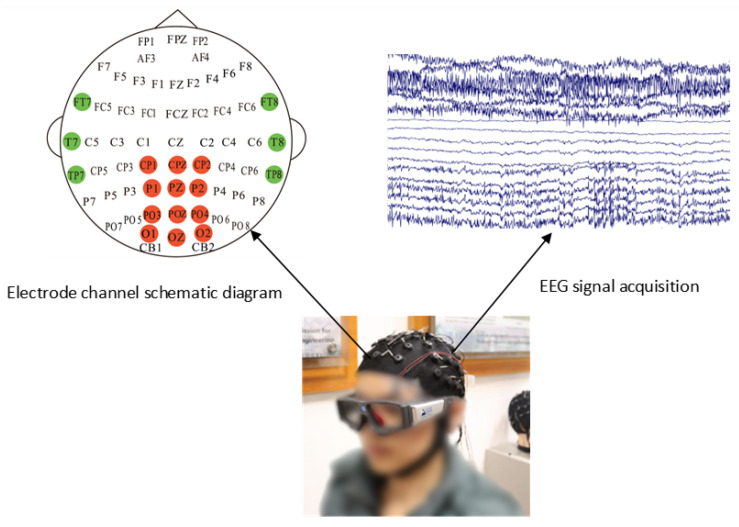
Schematic diagram of electrode channel and EEG signal acquisition.

The SEED-VIG database [32], a benchmark for driver fatigue studies, was adopted in this work. Developed by Shanghai Jiao Tong University’s Brain-Inspired Computing team, it includes data from 23 subjects (mean age: 23.3 years). Experiments were conducted in a virtual driving simulator with a projected road scene. The 118-min experimental sessions were primarily scheduled in the afternoon to facilitate fatigue induction. Participants wore EEG caps for electrophysiological signal acquisition and SMI gaze-tracking apparatus to measure eye movements, with PERCLOS values calculated for fatigue-level annotation. EEG recordings in the SEED-VIG dataset were obtained at 1000 Hz sampling rate from 17 standard channels referenced to CPZ, covering frontal to occipital areas (FT7, FT8, T7, T8, TP7, TP8, CP1, CP2, P1, PZ, P2, PO3, POZ, PO4, O1, OZ, O2). Figure 6 illustrates the schematic configuration of these 17 electrode positions.

The amplitude of EEG signals is at the microvolt level, making them highly susceptible to interference from various noise sources [33]. Consequently, the acquired data consists of both EEG signals and diverse noise components, with all non-EEG signals collectively referred to as artifacts [34]. The EEG signals were initially processed using a 1–75 Hz bandpass filter for noise suppression, with the sampling rate subsequently de-creased to 200 Hz to optimize computational efficiency. During the feature extraction stage, power spectral density (PSD) and DE features were extracted from EEG sig-nals across five frequency bands (δ: 1–4 Hz, θ: 4–8 Hz, α: 8–14 Hz, β: 14–31 Hz, and γ: 31–50 Hz). These features were computed using short-time Fourier transform (STFT) with non-overlapping 8-s windows. Additionally, features were extracted across the entire 1–50 Hz frequency band with a 2 Hz resolution. PSD reflects the energy distribution of EEG signals across different frequency bands, which has been demonstrated to be highly correlated with fatigue and drowsiness [35,36,37]. DE, characterizing the complexity and uncertainty of EEG signals, can be regarded as a complexity feature. Studies have shown that various entropy-based and complexity measurement methods can effectively identify fatigue states [38]. Notably, empirical evidence suggests that differential entropy features outperform power spectral density in fatigue detection [16].

One of the most significant manifestations of fatigue is reflected in the eyes [39], where the PERCLOS demonstrates a robust association with fatigue levels. PERCLOS is measured as the duration during which the eyelids cover the pupils within a given period, providing an objective metric for fatigue assessment with its computational formula given in Equation (17).

(17) $$\mathrm{PERCLOS}=\left(\frac{T_{\mathrm{closure}}}{T_{\mathrm{total}}}\right)\times 100\%$$

where $T_{\mathrm{closure}}$ is the eyelid closure time within the given time window, and $T_{\mathrm{total}}$ is the total observation time window. In the experiment, PERCLOS was computed over non-overlapping 8-s windows ($T_{\mathrm{total}}=8$ s), generating 885 time steps in total. The PERCLOS metric is bounded by 0 and 1, where elevated scores correspond to increased fatigue degree. Based on reference [32], the fatigue states were classified as: alert [0, 0.35], fatigued [0.35, 0.7], and drowsy [0.7, 1]. Although PERCLOS is often discretized into two or three levels in prior studies [40,41,42], this study treats it as a continuous variable to preserve subtle temporal dynamics and enable fine-grained prediction. The regression approach facilitates proactive fatigue degree forecasting rather than post hoc state recognition.

In this study, we further analyzed the data distribution of PERCLOS values in the SEED-VIG dataset. The samples are distributed as follows: 36.32% in the alert range (0–0.35), 43.76% in the fatigued range (0.35–0.7), and 19.91% in the drowsy range (0.7–1.0). This distribution reveals a mild imbalance across fatigue levels, reflecting the natural evolution of fatigue during prolonged simulated driving. To mitigate potential bias caused by this imbalance, we conducted LOOCV experiments at the subject level, ensuring subject independence and preventing data leakage. Moreover, regression-based metrics (RMSE and MAE) were adopted, which are less sensitive to uneven class proportions compared with categorical accuracy, thereby maintaining fair evaluation across different fatigue levels.

### 4.2. Evaluation Metrics

Model performance was evaluated using both RMSE and MAE metrics, calculated as follows:

(18) $$\mathrm{RMSE}=\sqrt{\frac{1}{m}\sum_{i=1}^{m}{\left(y_{i}-{\hat{y}}_{i}\right)}^{2}}$$

(19) $$\mathrm{MAE}=\frac{1}{m}\sum_{i=1}^{m}\left|y_{i}-{\hat{y}}_{i}\right|$$

In the above, $y_{i}$ is the true value, ${\hat{y}}_{i}$ is the predicted value, and *m* is the number of samples.

The RMSE measures the square root of the average squared differences between predicted and actual values. It penalizes larger errors more heavily, making it particularly sensitive to outliers. Therefore, RMSE reflects the overall deviation magnitude of the model predictions.

The MAE represents the average of the absolute differences between predicted and actual values. It provides a more intuitive interpretation of prediction accuracy, as it directly reflects the average prediction deviation without disproportionately amplifying large errors.

In summary, MAE indicates the average level of prediction error, while RMSE emphasizes the influence of large errors. Using both metrics together provides a comprehensive evaluation of the prediction performance.

### 4.3. Implementation Details

#### 4.3.1. LOOCV Experiment

**Figure 7:**
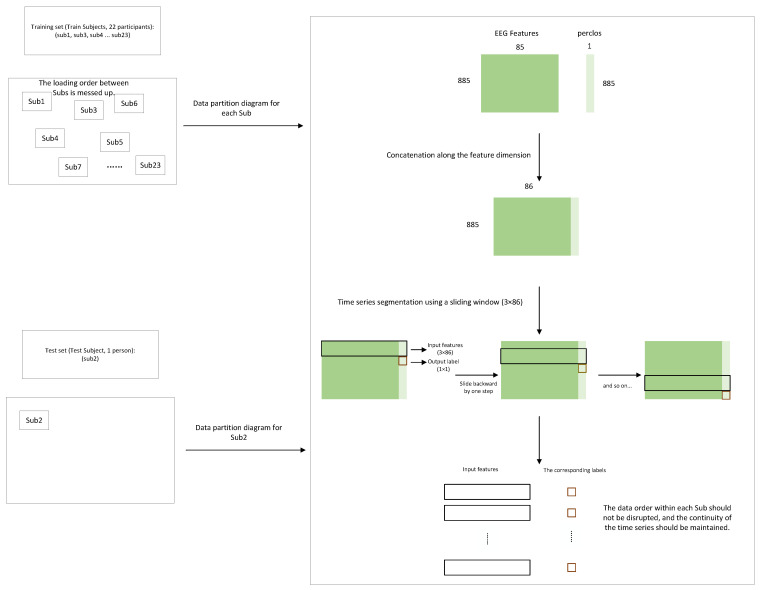
Schematic diagram of dataset partitioning in the LOOCV experiment (using sub2 as the test set example).

The LOOCV strategy was adopted to evaluate the model’s generalization performance across subjects. The core concept is that each time one subject is selected as the test set, while all remaining subjects form the training set. This process is repeated until every subject has served as the test set once, and finally, all test results are aggregated for comprehensive evaluation. Subject order is shuffled across participants during training, with within-subject temporal continuity strictly maintained. The data partitioning scheme is illustrated in Figure 7.

#### 4.3.2. Cross-Subject Pre-Training with Within-Subject Fine-Tuning Experiment

The experiment adopts a two-stage training strategy, consisting of a pre-training stage and a fine-tuning stage. This study falls within the scope of transfer learning, which has demonstrated exceptional cross-task and cross-domain adaptation capabilities across multiple fields including computer vision, natural language processing, and biomedical signal analysis, with its effectiveness being extensively validated [43,44,45,46,47,48].

During the pre-training stage, the training sets of all 23 samples are combined into a unified pre-training dataset, and the order of the training sets is randomly shuffled each epoch while preserving the internal temporal sequence of each sample. Early stopping is monitored simultaneously using the validation sets of all 23 samples during pre-training. In the fine-tuning stage, the pre-trained weights obtained from the pre-training stage are used to fine-tune the parameters for individual samples, with each sample’s training set utilized for fine-tuning. Early stopping is monitored using the respective sample’s validation set, and the final evaluation is performed on the sample’s test set. This cross-subject to within-subject hybrid design enables simultaneous learning of population-level common features and adaptation to individual variations.

This study constructs an EEG dataset by concatenating DE and eyelid closure degree at the feature level. Let the differential entropy matrix be $X_{DE}\in R^{885\times 85}$, the eyelid closure vector be $y\in R^{885\times 1}$, and the concatenated dataset be $D=\left[X_{DE},y\right]\in R^{885\times 86}$. Subsequently, a sliding window is constructed for the dataset $D\in R^{885\times 86}$, using the data from the previous three time steps to predict the PERCLOS value at the next time step. Time step 3 is the optimal solution obtained through comparative experiments (Section 4.5.1). The 23 time-series data samples are split into training, validation, and test sets in an 8:1:1 ratio, without shuffling the temporal order. The training set is used for model training, the validation set for monitoring the training process in combination with an early-stopping strategy, and the test set for the final evaluation of model performance. For each *i* in the training, validation, and test sets:

(20) $$X_{i}=\left[\begin{array}{c} D[i]\\ D[i+1]\\ D[i+2] \end{array}\right]\in R^{3\times 86},\;y_{i}=D\left[i+3,86\right]\in R$$

To eliminate dimensional and scale differences while accelerating model convergence, this study processes the data using Z-score standardization, scaling the data to follow a standard normal distribution. The calculation formula is as follows:

(21) $$z=\frac{x-\mu }{\sigma }$$

μ corresponds to the mean, and σ to the standard deviation.

#### 4.3.3. Additional Feature Extraction

To further explore the discriminative patterns in EEG signals, we extracted three additional features from the raw EEG data in the SEED-VIG dataset: the α/β band power ratio, wavelet entropy, and Hurst exponent. Prior to feature extraction, the raw EEG data underwent preprocessing involving 1–75 Hz bandpass filtering and artifact removal through Independent Component Analysis (ICA). These procedures were implemented using the EEGLAB toolbox, a MATLAB (version R2021a) toolkit specifically designed for EEG signal processing.

**Figure 8:**
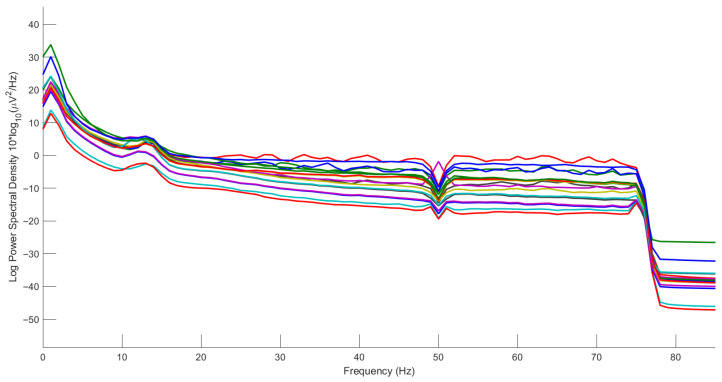
Power spectral density after 1–75 Hz filtering.

Filtering effectively isolates fatigue-related frequency bands and suppresses signal interference. As shown in Figure 8, the filtered power spectral density exhibits significant attenuation beyond 75 Hz.

**Figure 9:**
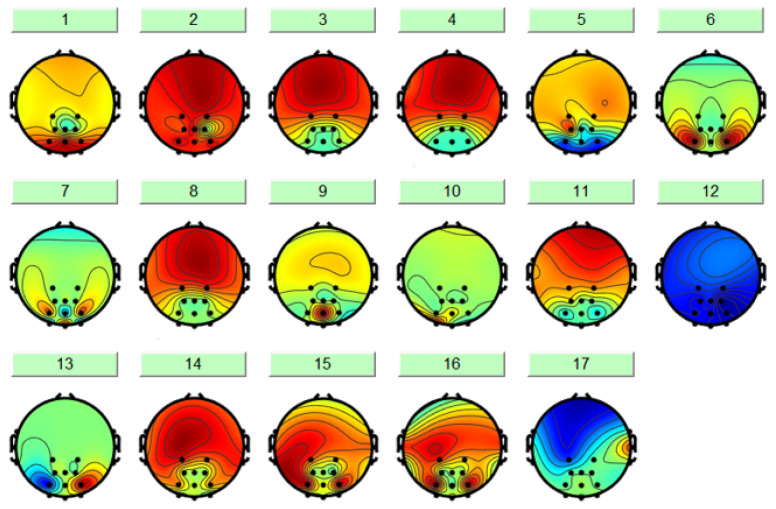
17 ICA components.

ICA is an effective method for blind source separation [49], which enables the isolation of interference signals unrelated to fatigue, such as EOG and electromyographic (EMG) signals. As illus-trated in Figure 9, the ICA-derived results comprise 17 components, among which some represent genuine EEG signals while others constitute artifacts. For subsequent analysis, it is essential to remove these artifactual components while retaining the purified EEG signals.

**Figure 10:**
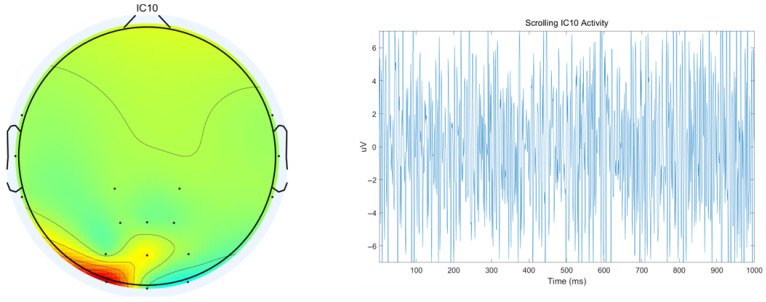
EMG artifact-related components.

A representative example of artifacts is shown in Figure 10, where EMG signals are illustrated-with the left panel displaying the topographic brain map and the right panel presenting the time-domain representation of EMG artifacts.

**Figure 11:**
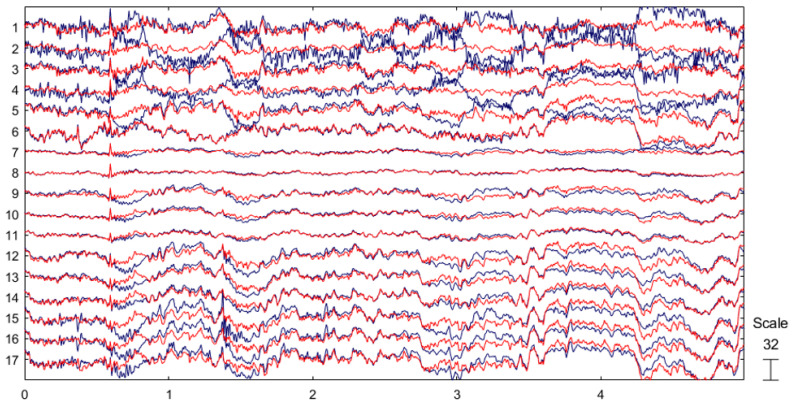
Comparison of EEG signals before and after preprocessing After preprocessing the raw EEG signals to obtain.

A comparison between the raw EEG signals and preprocessed EEG signals is shown in Figure 11, where the blue waveforms represent the original EEG data and the red waveforms denote the preprocessed EEG signals.

After preprocessing the raw EEG signals to obtain clean electrophysiological data, we subsequently extracted three key EEG features for analysis: the α/β band power ratio, wavelet entropy, and Hurst exponent. Below we provide a brief introduction to each of these features.

Band power ratio, a commonly used quantitative metric in EEG signal analysis, reflects the activity intensity of different frequency bands. Typically, EEG signals are divided into five frequency bands. The α band is associated with relaxation and eye-closed states, while the β band correlates with concentration and cognitive activities. A high α/β ratio is commonly observed during relaxed or meditative states. An increased α/β ratio indicates mental fatigue. This ratio is calculated as the power ratio between the α and β bands. In this study, the α/β ratio was computed using STFT with non-overlapping 8-s windows. The formula for calculating the α/β band power ratio is as follows:

(22) $$\alpha /\beta \;Ratio=\frac{P_{\alpha }}{P_{\beta }}$$

(23) $$P_{\alpha }=\sum_{f\;=\;8\;\mathrm{Hz}}^{14\;\mathrm{Hz}}\mathrm{PSD}\left(f\right)$$

(24) $$P_{\beta }=\sum_{f\;=\;14\;\mathrm{Hz}}^{31\;\mathrm{Hz}}\mathrm{PSD}\left(f\right)$$

Wavelet Entropy [50] is a signal analysis method that combines wavelet transform and information entropy to quantify signal complexity. The wavelet entropy value decreases when the EEG signal exhibits regular and ordered patterns, whereas it increases when the signal becomes complex and disordered. To compute wavelet entropy, the signal is first decomposed using wavelet transform to obtain wavelet coefficients $W_{j}\left(t\right)$ at different scales. The energy $E_{j}$ at each scale j is then calculated, followed by the determination of relative energy $p_{j}$. Finally, wavelet entropy is derived using Shannon’s entropy formula as follows:

(25) $$E_{j}=\sum_{t}|W_{j}\left(t\right){|}^{2}$$

(26) $$p_{j}=\frac{E_{j}}{\sum_{k}E_{k}}$$

(27) $$WE=-\sum_{j}p_{j}\cdot \log\left(p_{j}\right)$$

This study employs non-overlapping 8-s windows for wavelet transformation using db4 (Daubechies 4) as the mother wavelet with a 5-level decomposition. The δ, θ, α, β, and γ frequency bands were extracted, and wavelet entropy was subsequently calculated based on these five frequency bands.

The Hurst exponent was originally proposed by hydrologist Harold Hurst [51] and has since been widely applied across various disciplines. This metric quantifies the long-range dependence or self-similarity of EEG signals, thereby reflecting the complexity of brain activity. During fatigue states, the complexity of neural activity decreases while the regularity of EEG signals increases—changes that can be effectively captured by the Hurst exponent. In this study, we performed 5-level decomposition using db4 wavelets for each 8-second non-overlapping window per channel. The reconstructed signals from each frequency band were subsequently analyzed using the rescaled range (R/S) analysis to compute their respective Hurst exponents. The calculation formula for the Hurst exponent is as follows:

(28) $$H=\frac{\log\left(R/S\right)}{\log\left(T\right)}$$

The formula parameters are defined as follows: *R* represents the rescaled range, *S* denotes the standard deviation of the time series, *T* indicates the time span (number of data points) of the series, and *H* is the Hurst exponent whose value typically ranges between 0≤H≤1.

### 4.4. Training Settings

#### 4.4.1. LOOCV Experiment

The experiment was conducted using the PyTorch (1.11.0) framework on an NVIDIA RTX 3050 GPU. During the training process, the MSE loss function was used, with the Adam [52] optimizer, an initial learning rate of 0.001, a batch size of 64, and a fixed number of 20 training epochs.

To ensure reproducibility, all experiments were conducted with fixed random seeds (refer to the hyperparameter settings in Table A1). All model weights were initialized using PyTorch’s default schemes (Kaiming uniform for convolutional layers and Xavier uniform for linear layers). The Z-score normalization described in Equation (21) was applied, where the mean and standard deviation were computed from the training set only to avoid data leakage.

#### 4.4.2. Cross-Subject Pre-Training with Within-Subject Fine-Tuning Experiment

In the pre-training phase, the Adam optimizer was used with an initial learning rate of 0.001, and the learning rate was dynamically adjusted—halved if the validation loss did not decrease for 5 epochs. The total number of training epochs was 150, and an early stopping mechanism was employed, terminating the training if the validation loss showed no improvement for 10 epochs. The batch size was set to 64. Using the MSE loss function. In the fine-tuning phase, the model was trained for 50 epochs with a learning rate of 0.0001, while all other training configurations remained consistent with those used in the pre-training phase.

### 4.5. Main Results

#### 4.5.1. Window Length Experiment

redAs shown in Table 2, from the average results (Avg), a window length of 3 performed the best (RMSE = 0.0598, MAE = 0.0509), with the lowest error. This indicates that for fatigue detection based on EEG (DE features) + PERCLOS, selecting an appropriate time window can significantly improve the model’s prediction accuracy.

#### 4.5.2. Comparison of Univariate and Multimodal Fatigue Prediction Based on LSTM

This study employs an LSTM model for fatigue degree prediction and designs two comparative experiments based on a LOOCV paradigm: the first group uses univariate modeling based solely on PERCLOS (PERCLOS-only), while the second group employs multimodal fusion modeling combining EEG signals (DE features) and PERCLOS (EEG + PERCLOS). As shown in Table 3, the prediction performance of the multimodal fusion model (Avg RMSE = 0.0598) is significantly better than that of the univariate model (Avg RMSE = 0.0683), with a relative error reduction of 12.4%. These results confirm the effectiveness of multimodal physiological signal fusion in fatigue degree prediction, demonstrating that EEG features and PERCLOS indicators exhibit complementarity, and their joint modeling can improve prediction accuracy.

#### 4.5.3. Ablation Study

To rigorously evaluate the contribution of each architectural component in CTL-ResFNet, we conducted a systematic ablation study based on a module-removal paradigm. The experiments were performed using Leave-One-Out Cross-Validation (LOOCV) on the dataset, utilizing Differential Entropy (DE) as the input feature. We compared the complete CTL-ResFNet against four incomplete variants:

**-CNN**: Removing the Convolutional Neural Network module to test the importance of local feature extraction.
**-Transformer**: Removing the Transformer module to evaluate the impact of the self-attention mechanism.
**-LSTM**: Removing the Long Short-Term Memory network to assess the necessity of temporal dynamic modeling.
**-Residual**: Removing the residual connections to verify their role in feature fusion and training stability.

The quantitative results of these experiments are summarized in Table 4.

As evidenced by the average metrics in Table 4, the complete CTL-ResFNet architecture achieves the best performance with the lowest RMSE (0.0266) and MAE (0.0215). The removal of any single component leads to varying degrees of performance degradation, confirming that all designed modules contribute positively to the model’s predictive capability.

**Impact of Residual Connections.** The most significant performance drop occurs in the -Residual variant, where the average RMSE surges to 0.0692 and MAE to 0.0549. This stark contrast suggests that residual connections are critical for the network. They likely facilitate efficient gradient flow and prevent information loss during the integration of features from different layers, ensuring the model can effectively learn complex mappings without degradation.

**Impact of Temporal Modules (LSTM).** The -LSTM variant shows the second-highest error rates (RMSE: 0.0531). Since EEG signals are inherently time-series data, this result underscores the importance of the LSTM module in capturing long-term temporal dependencies. Without LSTM, the model fails to adequately account for the temporal dynamics and historical context of the brain signals.

**Impact of Feature Extraction (CNN and Transformer).** Removing the feature extraction modules also negatively impacts performance, though to a lesser extent than the structural components above. The -CNN variant yields an RMSE of 0.0449, indicating that the CNN is essential for extracting local spatial-spectral features from the DE entropy maps.Meanwhile, the -Transformer variant results in an RMSE of 0.0331. While this is the smallest drop among the variants, it is still notably higher than the full model. This demonstrates that the Transformer’s self-attention mechanism provides a valuable refinement by capturing global dependencies and weighting critical feature segments, thereby enhancing the final prediction accuracy.

In conclusion, the ablation study validates the rationale behind CTL-ResFNet: the CNN extracts local features, the Transformer refines global attention, the LSTM captures temporal evolution, and the residual connections ensure stable integration, all working synergistically to achieve optimal performance.

#### 4.5.4. Cross-Subject Generalization and Individual Adaptation Analysis

This study systematically validated the superior performance of the CTL-ResFNet model through LOOCV experiments and pretraining–finetuning comparison experiments. The experiments selected CNN, Transformer, LSTM, and CNN-Transformer as baseline models for comparative analysis. As shown in Table 5, in the LOOCV, CTL-ResFNet achieved an average RMSE of 0.0266 and an average MAE of 0.0215, significantly outperforming all comparative models. This result fully demonstrates the model’s exceptional generalization capability in zero-shot cross-subject scenarios, highlighting its unique architectural advantages. Further fine-tuning experimental results (Table 6) revealed that CTL-ResFNet (Avg RMSE = 0.0935) also exhibited significant advantages in individual adaptation ability, further validating the model’s strong transferability and adaptability.

**Figure 12:**
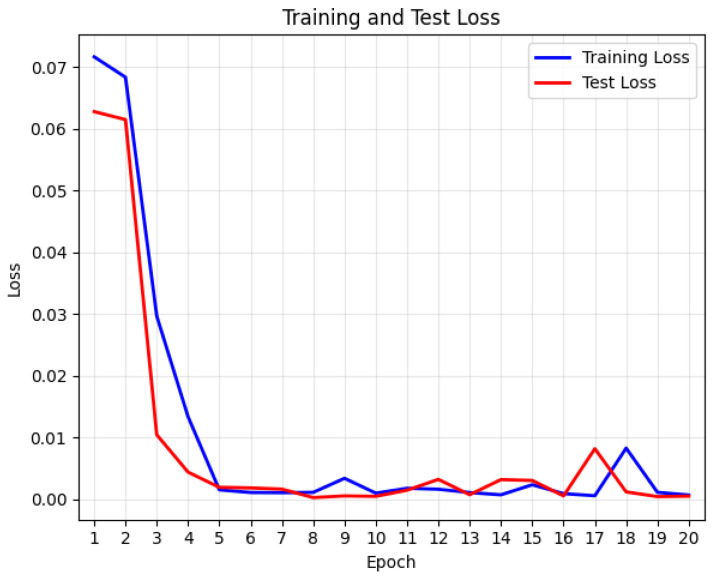
Training curve, taking sub2 as an example.

**Figure 13:**
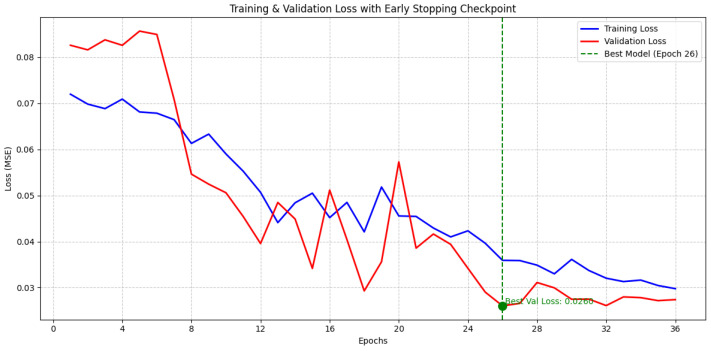
The pre-training process training curve, where the point with the lowest validation set loss is the moment when the early stopping mechanism saves the model.

The training curves of the experiments are shown in Figure 12 and Figure 13.

#### 4.5.5. Comparison of Feature Performance Between LOOCV and Fine-Tuning Experiments

This study further extracted three feature indicators from EEG signals: the α/β band power ratio, wavelet entropy, and Hurst exponent. Two experimental paradigms—LOOCV and pretraining–finetuning (based on the CTL-ResFNet framework)—were employed for validation. As shown in Table 7, under the LOOCV setting, the α/β band power ratio demonstrated the best predictive performance, achieving an average RMSE of 0.0190. In contrast, in the pretraining–finetuning experiments presented in Table 8, the DE feature performed the best, with an average RMSE of 0.0935. This comparative result indicates that the optimal feature selection varies significantly under different validation paradigms, suggesting that the most suitable feature indicators should be chosen based on the specific experimental design in practical applications.

To evaluate the feasibility of deploying CTL-ResFNet in real-time fatigue monitoring scenarios, we further measured the inference speed on different hardware platforms. On a standard laptop equipped with an AMD R7-6800H CPU (Advanced Micro Devices, Inc., Santa Clara, CA, USA), the average inference time per sample was 2.022 ms, corresponding to a throughput of approximately 495 samples per second. On an embedded NVIDIA Jetson Nano B01 device (NVIDIA Corporation, Santa Clara, CA, USA), the average inference time per sample was 8.578 ms (≈117 samples/s). These results demonstrate that CTL-ResFNet can operate in real-time even under limited computational resources. The lightweight design effectively balances temporal modeling capacity and computational efficiency, making the model suitable for integration into embedded automotive fatigue-monitoring systems.

## 5. Conclusions

This paper introduces CTL-ResFNet, a novel deep learning model that integrates CNN, TransformerEncoder, LSTM, and residual connections to predict driver fatigue degree using historical EEG signals and PERCLOS. By effectively capturing spatiotemporal dependencies in EEG data, the proposed model outperforms baseline approaches (CNN, Transformer, LSTM, and CNN-Transformer) in both LOOCV and pretraining–finetuning experiments, as reflected by lower RMSE and MAE values.

Results demonstrate that combining EEG-based DE features with PERCLOS substantially improves fatigue degree prediction performance compared to using PERCLOS alone, as confirmed through LSTM validation experiments. Comparative analysis of alternative EEG features—including the α/β band power ratio, wavelet entropy, and Hurst exponent—reveals that while the α/β ratio performs best in cross-validation, DE features exhibit superior effectiveness in pretraining–finetuning scenarios, highlighting the need for context-aware feature selection.

These findings emphasize the promise of multimodal deep learning for fatigue monitoring, with CTL-ResFNet providing a robust framework for real-world deployment. Future research directions could include enhancing model interpretability through attention-based visualization or explainable AI methods such as SHAP or Grad-CAM to analyze which EEG channels and features contribute most to fatigue prediction. In addition, since the SEED-VIG dataset used in this study involves participants with a relatively narrow age range and unspecified gender distribution, the effects of demographic factors such as age and gender on fatigue prediction were not explicitly examined. Future work will therefore expand the dataset to include participants of diverse ages and genders to explore demographic influences on fatigue modeling and improve the generalizability of the proposed method.

## Figures and Tables

**Table 1:** CTL-ResFNet Parameter Information.

Component	Parameters
PositionalEncoding	d_model = 86
Conv1d	input = 86, output = 64, kernel = 1
TransformerEncoderLayer	d_model = 64, nhead = 4, dim_feedforward = 64
TransformerEncoder	1 layer
LSTM	input = 64, hidden = 32, layers = 2
FC	input = 32, output = 1

**Table 2:** Experimental Study on Window Length Selection Based on LSTM (LOOCV Experiment, EEG + PERCLOS).

Sub.	Window Length 1		Window Length 2		Window Length 3		Window Length 4		Window Length 5	
	RMSE	MAE	RMSE	MAE	RMSE	MAE	RMSE	MAE	RMSE	MAE
1	0.1154	0.1004	0.1131	0.0974	0.0768	0.0652	0.0793	0.0711	0.0733	0.0630
2	0.0493	0.0393	0.0481	0.0414	0.0356	0.0302	0.0830	0.0723	0.0283	0.0213
3	0.0861	0.0767	0.0778	0.0728	0.0469	0.0407	0.0523	0.0489	0.0935	0.0870
4	0.0531	0.0433	0.1000	0.0844	0.0443	0.0371	0.0508	0.0417	0.0754	0.0664
5	0.0572	0.0468	0.0636	0.0564	0.0730	0.0651	0.0710	0.0619	0.0817	0.0724
6	0.0888	0.0659	0.0615	0.0488	0.0582	0.0429	0.0625	0.0467	0.0741	0.0611
7	0.1650	0.1161	0.1764	0.1170	0.0526	0.0445	0.1352	0.1010	0.0910	0.0698
8	0.0580	0.0479	0.0305	0.0249	0.0672	0.0622	0.0463	0.0436	0.0524	0.0492
9	0.1202	0.1091	0.1809	0.1655	0.0566	0.0538	0.0378	0.0327	0.0549	0.0518
10	0.0343	0.0276	0.0413	0.0319	0.0545	0.0411	0.0351	0.0269	0.0377	0.0295
11	0.0235	0.0183	0.0418	0.0323	0.0365	0.0281	0.0474	0.0439	0.0498	0.0467
12	0.0486	0.0440	0.0438	0.0393	0.0451	0.0394	0.0474	0.0435	0.0482	0.0448
13	0.0321	0.0283	0.0384	0.0307	0.0707	0.0649	0.0622	0.0578	0.0822	0.0750
14	0.0828	0.0675	0.0843	0.0689	0.0692	0.0566	0.0635	0.0512	0.0637	0.0520
15	0.1075	0.0990	0.0575	0.0511	0.0706	0.0607	0.0499	0.0425	0.0684	0.0604
16	0.0677	0.0567	0.0719	0.0600	0.0497	0.0433	0.0464	0.0372	0.0591	0.0510
17	0.0555	0.0502	0.0442	0.0377	0.0483	0.0438	0.0547	0.0493	0.0466	0.0416
18	0.0600	0.0490	0.1009	0.0876	0.0789	0.0704	0.0687	0.0602	0.0698	0.0621
19	0.0573	0.0446	0.0573	0.0495	0.0709	0.0596	0.0627	0.0501	0.0668	0.0559
20	0.0918	0.0719	0.0927	0.0713	0.0827	0.0688	0.0619	0.0531	0.0569	0.0482
21	0.0365	0.0336	0.0436	0.0339	0.0581	0.0522	0.1008	0.0791	0.0389	0.0363
22	0.0759	0.0628	0.0719	0.0491	0.0528	0.0425	0.0553	0.0414	0.0746	0.0573
23	0.0821	0.0711	0.0605	0.0506	0.0764	0.0574	0.0495	0.0412	0.0559	0.0435
Avg.	0.0717	0.0596	0.0740	0.0609	**0.0598**	**0.0509**	0.0619	0.0521	0.0627	0.0542

**Table 3:** Experimental Comparison of LSTM-based EEG + PERCLOS and PERCLOS-only Approaches (LOOCV Experiment).

Sub.	EEG + PERCLOS		PERCLOS-Only	
	RMSE	MAE	RMSE	MAE
1	0.0768	0.0652	0.0842	0.0767
2	0.0356	0.0302	0.0399	0.0335
3	0.0469	0.0407	0.0595	0.0557
4	0.0443	0.0371	0.0465	0.0390
5	0.0730	0.0651	0.0672	0.0575
6	0.0582	0.0429	0.0852	0.0644
7	0.0526	0.0445	0.0564	0.0462
8	0.0672	0.0622	0.0491	0.0458
9	0.0566	0.0538	0.0520	0.0496
10	0.0545	0.0411	0.0592	0.0437
11	0.0365	0.0281	0.0537	0.0502
12	0.0451	0.0394	0.0542	0.0496
13	0.0707	0.0649	0.0698	0.0656
14	0.0692	0.0566	0.0866	0.0703
15	0.0706	0.0607	0.0861	0.0813
16	0.0497	0.0433	0.0640	0.0541
17	0.0483	0.0438	0.0464	0.0416
18	0.0789	0.0704	0.0802	0.0700
19	0.0709	0.0596	0.0853	0.0776
20	0.0827	0.0688	0.1028	0.0843
21	0.0581	0.0522	0.0706	0.0677
22	0.0528	0.0425	0.0786	0.0492
23	0.0764	0.0574	0.0941	0.0581
Avg.	**0.0598**	**0.0509**	0.0683	0.0579

**Table 4:** Results of ablation experiments (LOOCV experiment).

Sub.	CTL-ResFNet		-CNN		-Transformer		-LSTM		-Residual	
	RMSE	MAE	RMSE	MAE	RMSE	MAE	RMSE	MAE	RMSE	MAE
1	0.0411	0.0362	0.0421	0.0344	0.0369	0.0322	0.0779	0.0604	0.0251	0.0207
2	0.0257	0.0222	0.0403	0.0358	0.0539	0.0482	0.0350	0.0302	0.0807	0.0694
3	0.0136	0.0110	0.0252	0.0226	0.0551	0.0489	0.0825	0.0751	0.0612	0.0426
4	0.0401	0.0302	0.0314	0.0254	0.0288	0.0236	0.0335	0.0254	0.0446	0.0337
5	0.0256	0.0209	0.0381	0.0314	0.0193	0.0155	0.0360	0.0304	0.1461	0.1168
6	0.0332	0.0255	0.0350	0.0280	0.0315	0.0226	0.0374	0.0280	0.0743	0.0555
7	0.0261	0.0153	0.0487	0.0383	0.1290	0.0934	0.0347	0.0275	0.0681	0.0537
8	0.0231	0.0204	0.0590	0.0523	0.0470	0.0400	0.0315	0.0240	0.0731	0.0562
9	0.0215	0.0177	0.0589	0.0517	0.0541	0.0482	0.0812	0.0733	0.0776	0.0675
10	0.0194	0.0165	0.0401	0.0333	0.0356	0.0269	0.0332	0.0252	0.1121	0.0959
11	0.0415	0.0380	0.0496	0.0437	0.0171	0.0148	0.0478	0.0397	0.0482	0.0389
12	0.0158	0.0128	0.1085	0.0925	0.0216	0.0180	0.1245	0.1022	0.0446	0.0341
13	0.0160	0.0128	0.0296	0.0249	0.0137	0.0107	0.0398	0.0347	0.0772	0.0657
14	0.0283	0.0263	0.0584	0.0493	0.0216	0.0191	0.0798	0.0734	0.0966	0.0738
15	0.0224	0.0180	0.0288	0.0227	0.0188	0.0155	0.0663	0.0484	0.0801	0.0537
16	0.0355	0.0239	0.0309	0.0221	0.0191	0.0152	0.0411	0.0307	0.0678	0.0470
17	0.0224	0.0171	0.0194	0.0171	0.0186	0.0153	0.0206	0.0141	0.0395	0.0311
18	0.0204	0.0171	0.0243	0.0188	0.0249	0.0184	0.0706	0.0603	0.0435	0.0357
19	0.0191	0.0154	0.0170	0.0137	0.0169	0.0146	0.0444	0.0257	0.0512	0.0360
20	0.0426	0.0311	0.1139	0.0662	0.0190	0.0160	0.0593	0.0485	0.1170	0.0902
21	0.0113	0.0082	0.0285	0.0172	0.0167	0.0139	0.0163	0.0131	0.0331	0.0290
22	0.0139	0.0115	0.0238	0.0172	0.0241	0.0194	0.0554	0.0512	0.0268	0.0215
23	0.0549	0.0471	0.0807	0.0735	0.0371	0.0320	0.0721	0.0627	0.1041	0.0935
Avg.	**0.0266**	**0.0215**	0.0449	0.0362	0.0331	0.0271	0.0531	0.0437	0.0692	0.0549

**Table 5:** LOOCV Evaluation Results for Each Model.

Sub.	CTL-ResFNet		CNN		Transformer		LSTM		CNN-Transformer	
	RMSE	MAE	RMSE	MAE	RMSE	MAE	RMSE	MAE	RMSE	MAE
1	0.0411	0.0362	0.0405	0.0315	0.0589	0.0489	0.0768	0.0652	0.0453	0.0400
2	0.0257	0.0222	0.0830	0.0715	0.0789	0.0706	0.0356	0.0302	0.0331	0.0289
3	0.0136	0.0110	0.0873	0.0543	0.0793	0.0519	0.0469	0.0407	0.0239	0.0203
4	0.0401	0.0302	0.1599	0.0786	0.0792	0.0566	0.0443	0.0371	0.0267	0.0209
5	0.0256	0.0209	0.0770	0.0649	0.1258	0.1045	0.0730	0.0651	0.0420	0.0338
6	0.0332	0.0255	0.1426	0.1195	0.0752	0.0573	0.0582	0.0429	0.0337	0.0272
7	0.0261	0.0153	0.4533	0.3604	0.1719	0.1279	0.0526	0.0445	0.0460	0.0390
8	0.0231	0.0204	0.0444	0.0331	0.0570	0.0466	0.0672	0.0622	0.0337	0.0275
9	0.0215	0.0177	0.0260	0.0186	0.0613	0.0478	0.0566	0.0538	0.0559	0.0494
10	0.0194	0.0165	0.0594	0.0515	0.0846	0.0640	0.0545	0.0411	0.0311	0.0232
11	0.0415	0.0380	0.0658	0.0571	0.0663	0.0505	0.0365	0.0281	0.0334	0.0293
12	0.0158	0.0128	0.0609	0.0457	0.1105	0.0763	0.0451	0.0394	0.0327	0.0274
13	0.0160	0.0128	0.0933	0.0269	0.0974	0.0815	0.0707	0.0649	0.0402	0.0340
14	0.0283	0.0263	0.1164	0.0905	0.1113	0.0930	0.0692	0.0566	0.0414	0.0341
15	0.0224	0.0180	0.1121	0.0945	0.1085	0.0847	0.0706	0.0607	0.0421	0.0356
16	0.0355	0.0239	0.1100	0.0804	0.0656	0.0525	0.0497	0.0433	0.0398	0.0299
17	0.0224	0.0171	0.0516	0.0399	0.0717	0.0600	0.0483	0.0438	0.0234	0.0182
18	0.0204	0.0171	0.1077	0.0889	0.1352	0.1165	0.0789	0.0704	0.0385	0.0313
19	0.0191	0.0154	0.0476	0.0390	0.1430	0.1301	0.0709	0.0596	0.0339	0.0267
20	0.0426	0.0311	0.2137	0.1666	0.1186	0.1010	0.0827	0.0688	0.0429	0.0329
21	0.0113	0.0082	0.1304	0.0702	0.0702	0.0460	0.0581	0.0522	0.0354	0.0282
22	0.0139	0.0115	0.1097	0.0904	0.1143	0.1012	0.0528	0.0425	0.0386	0.0326
23	0.0549	0.0471	0.1052	0.0910	0.0772	0.0650	0.0764	0.0574	0.0631	0.0569
Avg.	**0.0266**	**0.0215**	0.1062	0.0811	0.0940	0.0754	0.0598	0.0509	0.0381	0.0316

**Table 6:** Fine-Tuning Results of Each Model.

Sub.	CTL-ResFNet		CNN		Transformer		LSTM		CNN-Transformer	
	RMSE	MAE	RMSE	MAE	RMSE	MAE	RMSE	MAE	RMSE	MAE
1	0.0614	0.0575	0.2610	0.2535	0.1310	0.1103	0.2582	0.2568	0.0222	0.0203
2	0.0562	0.0463	0.0811	0.0707	0.1869	0.1822	0.0442	0.0357	0.1210	0.1128
3	0.0273	0.0234	0.0432	0.0362	0.0684	0.0571	0.0970	0.0925	0.0506	0.0470
4	0.0248	0.0207	0.0702	0.0618	0.1581	0.1448	0.0372	0.0300	0.0783	0.0692
5	0.1746	0.1570	0.1398	0.1190	0.1230	0.1080	0.2056	0.1797	0.1779	0.1571
6	0.1457	0.1382	0.3041	0.2758	0.1021	0.0918	0.3290	0.2918	0.1325	0.1248
7	0.0214	0.0170	0.0695	0.0604	0.1633	0.1239	0.0531	0.0318	0.0809	0.0638
8	0.0665	0.0529	0.0353	0.0284	0.0394	0.0314	0.0389	0.0325	0.0704	0.0617
9	0.0519	0.0352	0.0462	0.0381	0.0656	0.0425	0.0429	0.0354	0.0763	0.0566
10	0.2650	0.2406	0.2357	0.2272	0.2391	0.2228	0.2003	0.1892	0.2902	0.2312
11	0.0337	0.0302	0.0389	0.0308	0.1015	0.0945	0.0530	0.0466	0.0641	0.0552
12	0.0920	0.0827	0.0968	0.0739	0.0939	0.0787	0.0495	0.0426	0.1488	0.1311
13	0.0468	0.0357	0.0660	0.0552	0.0657	0.0579	0.1504	0.1451	0.0650	0.0516
14	0.2876	0.2601	0.4093	0.3695	0.2025	0.1783	0.2420	0.2182	0.4397	0.3945
15	0.1545	0.1545	0.1620	0.1494	0.0755	0.0521	0.1659	0.1632	0.0124	0.0118
16	0.1401	0.1099	0.1397	0.1182	0.1243	0.0996	0.1584	0.1210	0.1969	0.1514
17	0.0465	0.0405	0.0573	0.0454	0.0975	0.0850	0.0571	0.0458	0.0794	0.0728
18	0.1168	0.1157	0.3279	0.3212	0.1627	0.1425	0.3749	0.3714	0.2220	0.1951
19	0.0191	0.0165	0.0445	0.0369	0.0419	0.0320	0.0572	0.0505	0.0577	0.0466
20	0.0461	0.0413	0.1481	0.1364	0.0932	0.0796	0.0599	0.0475	0.1290	0.1198
21	0.0528	0.0486	0.0395	0.0340	0.0484	0.0414	0.0808	0.0783	0.0577	0.0504
22	0.0624	0.0589	0.0940	0.0855	0.0629	0.0494	0.0398	0.0308	0.0493	0.0451
23	0.1574	0.1519	0.3042	0.2809	0.1172	0.0971	0.2366	0.2201	0.1871	0.1056
Avg.	**0.0935**	**0.0841**	0.1398	0.1265	0.1115	0.0958	0.1310	0.1198	0.1221	0.1041

**Table 7:** Experimental Results for Different Dimensional Features (CTL-ResFNet, LOOCV).

Sub.	α/β Ratio		Wavelet Entropy		Hurst Exponent		DE	
	RMSE	MAE	RMSE	MAE	RMSE	MAE	RMSE	MAE
1	0.0156	0.0116	0.0167	0.0140	0.0218	0.0177	0.0411	0.0362
2	0.0118	0.0102	0.0174	0.0148	0.0222	0.0194	0.0257	0.0222
3	0.0191	0.0175	0.0161	0.0130	0.0171	0.0145	0.0136	0.0110
4	0.0125	0.0088	0.0116	0.0093	0.0128	0.0100	0.0401	0.0302
5	0.0307	0.0230	0.0280	0.0223	0.0337	0.0283	0.0256	0.0209
6	0.0195	0.0133	0.0241	0.0189	0.0312	0.0248	0.0332	0.0255
7	0.0124	0.0091	0.0169	0.0134	0.0109	0.0083	0.0261	0.0153
8	0.0101	0.0084	0.0419	0.0335	0.0172	0.0145	0.0231	0.0204
9	0.0084	0.0074	0.0771	0.0707	0.0400	0.0382	0.0215	0.0177
10	0.0152	0.0118	0.0163	0.0115	0.0177	0.0142	0.0194	0.0165
11	0.0234	0.0221	0.0203	0.0154	0.0128	0.0099	0.0415	0.0380
12	0.0154	0.0106	0.0239	0.0187	0.0199	0.0151	0.0158	0.0128
13	0.0109	0.0076	0.0149	0.0120	0.0126	0.0099	0.0160	0.0128
14	0.0212	0.0164	0.0240	0.0199	0.0214	0.0176	0.0283	0.0263
15	0.0274	0.0232	0.0144	0.0111	0.0166	0.0137	0.0224	0.0180
16	0.0171	0.0118	0.0166	0.0128	0.0136	0.0108	0.0355	0.0239
17	0.0209	0.0131	0.0270	0.0195	0.0099	0.0072	0.0224	0.0171
18	0.0210	0.0186	0.0148	0.0119	0.0277	0.0230	0.0204	0.0171
19	0.0163	0.0123	0.0182	0.0127	0.0247	0.0218	0.0191	0.0154
20	0.0196	0.0141	0.0196	0.0172	0.0244	0.0188	0.0426	0.0311
21	0.0080	0.0054	0.0106	0.0086	0.0123	0.0097	0.0113	0.0082
22	0.0429	0.0282	0.0156	0.0136	0.0134	0.0111	0.0139	0.0115
23	0.0379	0.0326	0.0400	0.0355	0.0336	0.0294	0.0549	0.0471
Avg.	**0.0190**	**0.0147**	0.0229	0.0187	0.0203	0.0168	0.0266	0.0215

**Table 8:** Experimental results for different dimensional features (based on CTL-ResFNet, pretraining–finetuning).

Sub.	α/β Band Power Ratio		Wavelet Entropy		Hurst Exponent		DE	
	RMSE	MAE	RMSE	MAE	RMSE	MAE	RMSE	MAE
1	0.0847	0.0846	0.0645	0.0645	0.0816	0.0811	0.0614	0.0575
2	0.0513	0.0434	0.0546	0.0465	0.0485	0.0401	0.0562	0.0463
3	0.0536	0.0480	0.0595	0.0465	0.0655	0.0686	0.0273	0.0234
4	0.0494	0.0414	0.0728	0.0618	0.0487	0.0388	0.0248	0.0207
5	0.1519	0.1287	0.2120	0.1889	0.1995	0.1711	0.1746	0.1570
6	0.1093	0.1014	0.1068	0.0982	0.1379	0.1234	0.1457	0.1382
7	0.0402	0.0328	0.0614	0.0514	0.0617	0.0492	0.0214	0.0170
8	0.0706	0.0604	0.1020	0.0832	0.0692	0.0567	0.0665	0.0529
9	0.1024	0.0671	0.1454	0.1019	0.0974	0.0740	0.0519	0.0352
10	0.2422	0.2215	0.3099	0.2672	0.2181	0.1942	0.2650	0.2406
11	0.0348	0.0290	0.0178	0.0144	0.0662	0.0595	0.0337	0.0302
12	0.0732	0.0647	0.0856	0.0712	0.1069	0.0920	0.0920	0.0827
13	0.0487	0.0401	0.0670	0.0562	0.0760	0.0657	0.0468	0.0357
14	0.3335	0.3112	0.3606	0.3035	0.2892	0.2423	0.2876	0.2601
15	0.1313	0.1313	0.1138	0.1127	0.0898	0.0878	0.1545	0.1545
16	0.1614	0.1286	0.1717	0.1394	0.1786	0.1370	0.1401	0.1099
17	0.0536	0.0482	0.0223	0.0178	0.0462	0.0420	0.0465	0.0405
18	0.0922	0.0908	0.0833	0.0820	0.0663	0.0643	0.1168	0.1157
19	0.0245	0.0201	0.0602	0.0547	0.0409	0.0335	0.0191	0.0165
20	0.0493	0.0457	0.0506	0.0472	0.0617	0.0560	0.0461	0.0413
21	0.0485	0.0406	0.0639	0.0530	0.0539	0.0435	0.0528	0.0486
22	0.0603	0.0561	0.0649	0.0612	0.1720	0.1661	0.0624	0.0589
23	0.1459	0.1332	0.1778	0.1618	0.1649	0.1345	0.1574	0.1519
Avg.	0.0971	0.0856	0.1099	0.0950	0.1061	0.0929	**0.0935**	**0.0841**

**Figure A1:**
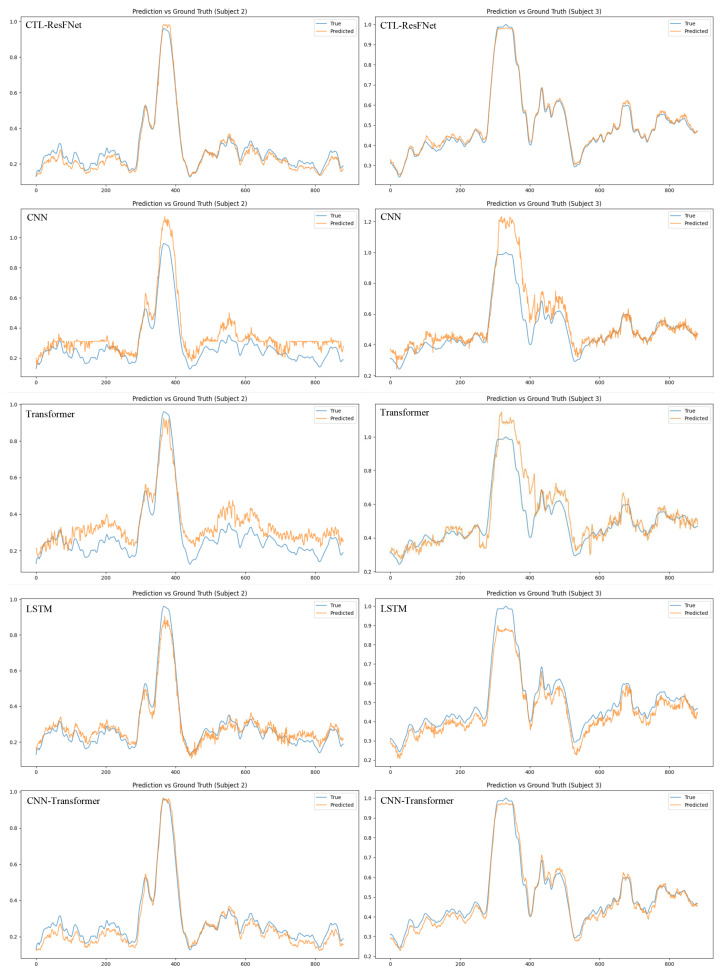
Prediction performance of each model in the LOOCV experiment, with the horizontal axis representing the time steps (a total of 885 time steps, each separated by 8 s) and the vertical axis representing PERCLOS.

**Figure A2:**
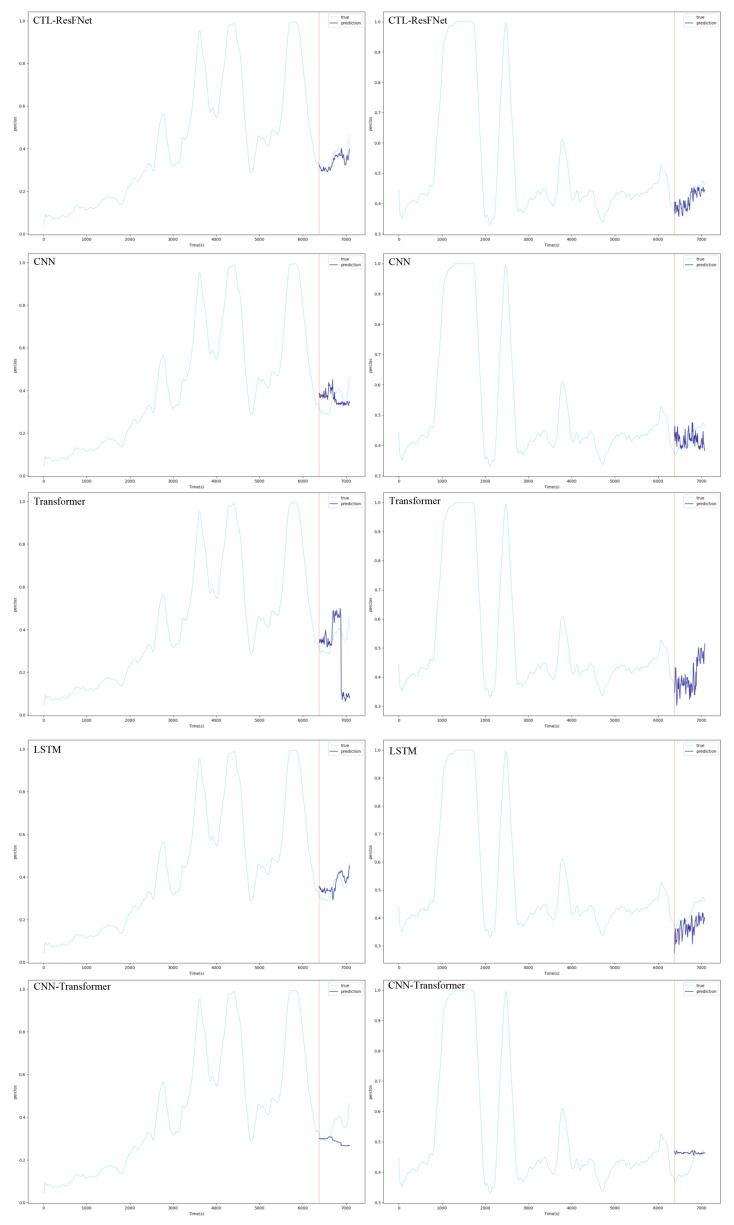
Prediction results of each model in the pre-training and fine-tuning experiment, with the x-axis representing time in seconds (total 7080 s) and the y-axis representing PERCLOS (Subject 7 (**left**) and Subject 19 (**right**)).

## Data Availability

The data analyzed in this study were sourced from the SEED-VIG dataset provided by Shanghai Jiao Tong University, accessible at: https://bcmi.sjtu.edu.cn/home/seed/seed-vig.html (accessed on 25 November 2025). This dataset is subject to access restrictions and requires submitting an application to the data provider for approval prior to use.

## Appendix A

### Appendix A.2

**Table A1:** Summary of hyperparameter settings under two experimental configurations.

Hyperparameter	LOOCV Default Value	Pretraining–Finetuning Default Value
Data Processing Related		
Sequence length	3	3
Validation set ratio	—	0.1
Test set ratio	—	0.1
Batch size	64	64
Normalization method	Z-Score normalization	Z-Score normalization
Model Architecture Related		
Batch size	64	64
Embedding dimension	64	64
LSTM hidden size	32	32
Number of LSTM layers	2	2
Number of Transformer heads	4	4
Transformer feedforward dimension	64	64
Transformer dropout rate	0.1	0.1
Convolution kernel size	1	1
Input feature dimension	86	86
Maximum positional encoding length	1000	1000
Positional encoding dropout rate	0	0
Training Strategy Related		
Optimizer type	Adam	Adam
Loss function	MSELoss	MSELoss
Learning rate	0.001	Pretraining: 0.001/Finetuning: 0.0001
Number of epochs	20	Pretraining: 150/Finetuning: 50
Learning rate scheduler	None	ReduceLROnPlateau
Scheduler decay factor	—	0.5
Scheduler patience	—	5
Early stopping	None	Enabled
Early stopping patience	—	10
Minimum improvement threshold	—	0.001
Randomness and Device Settings		
Random seed	42	42
Device	RTX3050	RTX3050
Environment Configuration		
Python version	3.9	3.9
CUDA version	11.3	11.3
PyTorch version	1.11.0	1.11.0

**Algorithm A1** Leave-One-Out Cross-Validation (LOOCV) Workflow for CTL-ResFNet
Require: Full Dataset $D=\{S_{1},S_{2},\ldots ,S_{23}\}$, where $S_{i}$ is the time-series data for subject *i*. Require: Hyperparameters: Window size w=3, Batch size B=64, Epochs E=20, Learning rate η=0.001. Ensure: Average performance metrics (RMSE, MAE) across all subjects. 1: **for** i←1 to 23 **do** ▹ Iterate through each subject as test set 2: $D_{test}\leftarrow S_{i}$ 3: $D_{train}\leftarrow D\setminus \left\{S_{i}\right\}$ 4: **Step 1: Sequence Generation (Sliding Window)** 5: $X_{train},Y_{train}\leftarrow \mathrm{SlidingWindow}\left(D_{train},w\right)$ ▹ Input *X*: 3 steps, Target *Y*: 4th step PERCLOS 6: $X_{test},Y_{test}\leftarrow \mathrm{SlidingWindow}\left(D_{test},w\right)$ 7: **Step 2: Z-score Normalization (Crucial: Prevent Data Leakage)** 8: ▹ Compute statistics solely on the aggregated training data 9: $\mu \leftarrow \mathrm{mean}(X_{train},\mathrm{axis}=0)$ 10: $\sigma \leftarrow \mathrm{std}(X_{train},\mathrm{axis}=0)$ 11: $X_{train}\leftarrow \left(X_{train}-\mu \right)/\left(\sigma +\epsilon \right)$ 12: $X_{test}\leftarrow \left(X_{test}-\mu \right)/\left(\sigma +\epsilon \right)$ ▹ Apply training stats to the test set 13: **Step 3: Data Loader Initialization** 14: $L_{train}\leftarrow \mathrm{GroupLoadersBySubject}\left(D_{train},B,\mathrm{shuffle}=\mathrm{False}\right)$ ▹ Each subject gets a separate DataLoader, preserving temporal order 15: $L_{test}\leftarrow \mathrm{DataLoader}\left(D_{test},B\right)$ 16: **Step 4: Model Training** 17: Initialize Model $M_{\theta }$ and Optimizer (Adam, η) 18: **for** epoch←1 to *E* **do** 19: $M_{\theta }.\mathrm{train}\left(\right)$ 20: **Shuffle** order of subject loaders in $L_{train}$ 21: **for** each subject_loader $L_{s}$ in $L_{train}$ **do** 22: **for** batch $\left(x_{b},y_{b}\right)$ in $L_{s}$ **do** 23: ${\hat{y}}_{b}\leftarrow M_{\theta }\left(x_{b}\right)$ 24: $L\leftarrow \mathrm{MSELoss}({\hat{y}}_{b},y_{b})$ 25: Backpropagate(L) 26: Optimizer.step() 27: **end for** 28: **end for** 29: **end for** 30: **Step 5: Evaluation** 31: $M_{\theta }.\mathrm{eval}\left(\right)$ 32: ${\hat{Y}}_{test}\leftarrow \mathrm{EvaluateModel}\left(M_{\theta },L_{test}\right)$ 33: Record RMSE and MAE between ${\hat{Y}}_{test}$ and $Y_{test}$ 34: **end for** 35: **return** Average RMSE and MAE across all LOOCV folds.

**Algorithm A2** Cross-Subject Pre-training with Within-Subject Fine-tuning
Require: All Subjects Data $D_{all}=\left\{S_{1},S_{2},\ldots ,S_{23}\right\}$. Require: Hyperparameters: Pre-train $\eta_{pre}=0.001$, Fine-tune $\eta_{ft}=0.0001$. Require: Early Stopping Patience P=10, LR Scheduler Patience $P_{lr}=5$. Ensure: Performance metrics on the test set of the target subject. 1: **Function ChronologicalSplitAndNormalize(**$S_{sub}$**)** ▹ Splits data $S_{sub}$ strictly by time and normalizes using training stats 2: $D^{tr},D^{val},D^{te}\leftarrow \mathrm{ChronologicalSplit}\left(S_{sub},\mathrm{ratios}=0.8,0.1,0.1\right)$ 3: $\mu ,\sigma \leftarrow \mathrm{Stats}(D^{tr})$ 4: $D^{tr},D^{val},D^{te}\leftarrow \mathrm{Normalize}\left(D^{tr},D^{val},D^{te},\mu ,\sigma \right)$ 5: **return** $L_{tr},L_{val},L_{te}$ (DataLoaders for each set) 6: **End Function** 7: **Phase 1: Cross-Subject Pre-training** 8: Initialize Model $M_{\theta }$, Optimizer $\left(\eta_{pre}\right)$, and Scheduler (ReduceLROnPlateau) 9: $L_{train\_all}\leftarrow \left[\right],L_{val\_all}\leftarrow \left[\right]$ 10: **for** i←1 to 23 **do** 11: $L_{tr}^{i},L_{val}^{i},L_{te}^{i}\leftarrow \mathrm{ChronologicalSplitAndNormalize}\left(S_{i}\right)$ ▹ Normalize each subject’s data independently 12: $L_{train\_all}.\mathrm{append}\left(L_{tr}^{i}\right)$ 13: $L_{val\_all}.\mathrm{append}\left(L_{val}^{i}\right)$ 14: **end for** 15: $\mathrm{ES}\_\mathrm{counter}\leftarrow 0,L_{min}\leftarrow \infty$ 16: **while** ES_counter<P **and** epoch<150 **do** 17: $\mathrm{Shuffle}\left(L_{train\_all}\right),\mathrm{Shuffle}\left(L_{val\_all}\right)$ ▹ Shuffle subject order, not sequence order within subject 18: $L_{train}\leftarrow \mathrm{AggregateTrainLoss}\left(M_{\theta },L_{train\_all}\right)$ 19: $L_{val}\leftarrow \mathrm{AggregateValidationLoss}\left(M_{\theta },L_{val\_all}\right)$ 20: $\mathrm{Scheduler}.\mathrm{step}(L_{val})$ 21: **if** $L_{val}<L_{min}$ **then** 22: $L_{min}\leftarrow L_{val}$, $\mathrm{Save}\;\mathrm{best}\;\theta_{pre}\leftarrow \theta$, ES_counter←0 23: **else** 24: ES_counter←ES_counter+1 25: **end if** 26: **end while** 27: Load $\theta \leftarrow \theta_{pre}$ 28: **Phase 2: Within-Subject Fine-tuning (Example: Target Subject *k*)** 29: k←1 ▹ Target subject 30: $L_{tr}^{k},L_{val}^{k},L_{te}^{k}\leftarrow \mathrm{ChronologicalSplitAndNormalize}\left(S_{k}\right)$ ▹ Re-split and Re-normalize for subject k 31: Initialize Optimizer $\left(\eta_{ft}=0.0001\right)$ and Scheduler (ReduceLROnPlateau) 32: $\mathrm{ES}\_\mathrm{counter}\leftarrow 0,L_{min}\leftarrow \infty$ 33: **while** ES_counter<P **and** epoch<50 **do** 34: $L_{train}^{k}\leftarrow \mathrm{TrainLoss}\left(M_{\theta },L_{tr}^{k}\right)$ 35: $L_{val}^{k}\leftarrow \mathrm{ValidationLoss}\left(M_{\theta },L_{val}^{k}\right)$ 36: $\mathrm{Scheduler}.\mathrm{step}(L_{val}^{k})$ 37: **if** $L_{val}^{k}<L_{min}$ **then** 38: $L_{min}\leftarrow L_{val}^{k}$, $\mathrm{Save}\;\mathrm{best}\;\theta_{ft}\leftarrow \theta$, ES_counter←0 39: **else** 40: ES_counter←ES_counter+1 41: **end if** 42: **end while** 43: Load $\theta \leftarrow \theta_{ft}$ ▹ Load best fine-tuned weights 44: ${\hat{Y}}_{test}^{k}\leftarrow \mathrm{EvaluateModel}\left(M_{\theta },L_{te}^{k}\right)$ 45: **return** RMSE and MAE on $S_{k}$’s test set.
